# Adaptation in U.S. Corn Belt increases resistance to soil carbon loss with climate change

**DOI:** 10.1038/s41598-020-70819-z

**Published:** 2020-08-14

**Authors:** Yao Zhang, Ernie Marx, Stephen Williams, Ram Gurung, Stephen Ogle, Radley Horton, Daniel Bader, Keith Paustian

**Affiliations:** 1 Natural Resource Ecology Laboratory, Colorado State University, Fort Collins, CO USA; 2 Department of Ecosystem Science and Sustainability, Colorado State University, Fort Collins, CO USA; 3 Center for Climate Systems Research, Columbia University, New York, NY USA; 4 Department of Soil and Crop Sciences, Colorado State University, Fort Collins, CO USA

**Keywords:** Agroecology, Carbon cycle, Climate-change mitigation

## Abstract

Increasing the amount of soil organic carbon (SOC) has agronomic benefits and the potential to mitigate climate change. Previous regional predictions of SOC trends under climate change often ignore or do not explicitly consider the effect of crop adaptation (i.e., changing planting dates and varieties). We used the DayCent biogeochemical model to examine the effect of adaptation on SOC for corn and soybean production in the U.S. Corn Belt using climate data from three models. Without adaptation, yields of both corn and soybean tended to decrease and the decomposition of SOC tended to increase leading to a loss of SOC with climate change compared to a baseline scenario with no climate change. With adaptation, the model predicted a substantially higher crop yield. The increase in yields and associated carbon input to the SOC pool counteracted the increased decomposition in the adaptation scenarios, leading to similar SOC stocks under different climate change scenarios. Consequently, we found that crop management adaptation to changing climatic conditions strengthen agroecosystem resistance to SOC loss. However, there are differences spatially in SOC trends. The northern part of the region is likely to gain SOC while the southern part of the region is predicted to lose SOC.

## Introduction

Soil organic matter is essential for maintaining soil health and sustaining plant growth. Loss of soil organic matter often leads to degradation of soil quality^1^. It also constitutes the largest terrestrial organic carbon (C) pool (~ 2,400 Pg C in top 2 m soil). This soil organic C (SOC) pool is three times greater than the amount of C in the atmosphere^2^. An increase or decrease of C in soils by only a small percent represents a substantial C sink or source for atmospheric CO_2_. Studies have been conducted to predict SOC of croplands under climate change^3–5^. However, crop management changes with adaptation to future climate^6–9^ (such as changes in varieties and planting dates) were ignored in most studies. Thus, we carried out a regional study to assess the impact of projected climate change and elevated CO_2_ on SOC in agricultural systems with management adaptation.

A change in SOC is a result of the net effect of the changes in SOC decomposition rates (the main C outflow) and C inputs from plants^10^ (main C inflow). In a warmer climate, the higher temperature could increase the decomposition rate in both the short and long term^11^. Decomposition will also be sensitive to increases or decreases in precipitation^10^ predicted by climate models^8^. Carbon input is mainly from root and surface residue litter that is not removed from the system through harvest, grazing or burning. Changes in temperature, precipitation, and atmospheric CO_2_ all affect this input through plant growth and production^6^. These climatic changes are also likely to affect management practices in the future^7^. Although systemic management changes are possible that can limit the impact of climate change, such as moving from dryland to irrigated systems, smaller adjustments including changes in varieties and planting dates have less barriers for adoption. These adjustments to management have been found to affect both crop production (C input) and decomposition^7,12^, with potentially larger effects on crop production^7^. These interactions are complex, and the overall change to SOC pools in agricultural lands remains uncertain.

Here we present the results from a simulation of SOC dynamics for more than 54,000 locations, covering 34,600,000 ha of cropland in the U.S. Corn Belt (Supplementary Fig. 1a). These locations have historically been managed with a corn (*Zea mays* L.) and soybean (*Glycine max* L.) rotation, which is the most common crop rotation in this region. Corn and soybean yields in this region account for about 85% of US crop production^13^. In our simulation, the widely used biogeochemical model DayCent^14,15^ was driven by weather data from the Representative Concentration Pathway 4.5 (RCP 4.5) climate change scenario^8^. Predictions from three General Circulation Models (GCMs; GFDL-CM3, MIROC-ESM, and MRI-CGCM3) were downscaled to generate daily weather (32 km grid) and used to assess the uncertainty. The main goal was to quantify the change in surface soil C (0–20 cm) under management adaptation with the selection of alternative crop varieties and planting dates to maintain high levels of crop production in future climate scenarios from 2041 to 2071. Alternative crop varieties were based on those currently available in the United States without consideration of additional crop breeding or genetic modifications that could further enhance production in the future. A no climate change scenario (historical weather with current CO_2_ level) was used as the baseline for comparisons.

### Crop production trends

**Figure 1:**
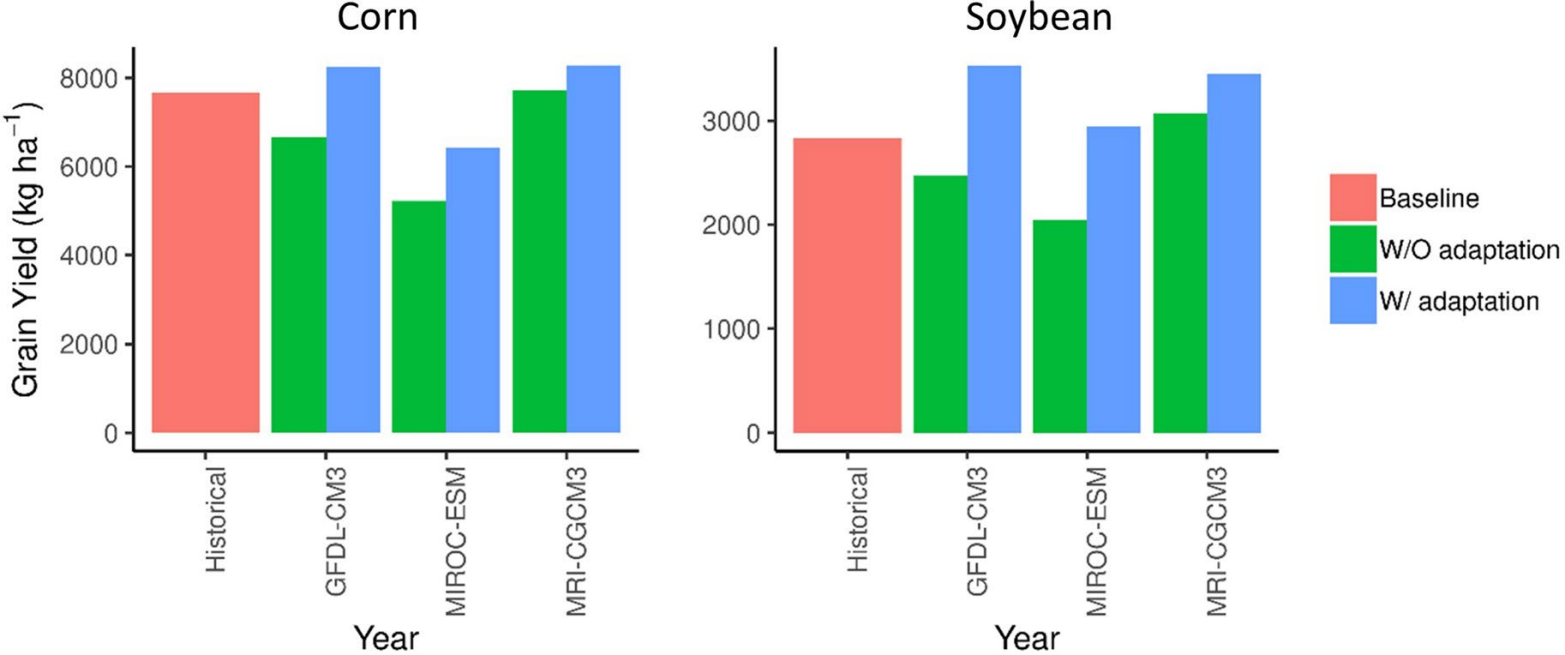
Predicted grain yield of (**a**) corn and (**b**) soybean in the 2041 to 2070 period.

We found that, without adaptation, the average corn yield during the 2041–2071 period in the Corn Belt would drop in all three GCM climate scenarios in comparison with the baseline with no climate change (Fig. 1). Yield decreased by 17% (GFDL-CM3), 34% (MIROC-ESM), and 2% (MRI-CGCM3) under the respective scenarios. The difference between GCMs can be attributed to differences in the projected temperature and precipitation (Supplementary Fig. 2). Similar yield decreases were seen for soybean as found for corn if there is no adaptation (decreased by 13% for GFDL-CM3, 28% for MIROC-ESM, and increased by 8% for MRI-CGCM3).

With adaptation, both corn and soybean yields increased compared with no adaptation: an increase of 5% for corn and 19% for soybean (average of the three GCMs) compared with the baseline. The larger increase in soybean yield was due to the C_3_ crop being more responsive to the CO_2_ fertilization than C_4_ crops^16^. The standard deviation of yields across the three GCMs in the adaptation scenarios was lower than that of the no adaptation scenarios (15% and 39% lower for corn and soybean respectively). Simulated crop yields were more stable under climate change with adaptation management compared to without adaptation. The stability in yields is because, without crop adaptation associated with the selection of alternative crop varieties, an increase in temperature shortens the growing period of the crop^17,18^. Each GCM predicts a longer growing season with warmer temperatures. When a longer-season maturity variety was simulated as an adaptation pathway, the extended growing period allows the crop to use the full window of optimal solar radiation. This leads to similar amounts of production from year to year between GCM climate scenarios. The spatial pattern of the yield changes was very different for the two crops (Supplementary Fig. 3) due to the difference in crop response to temperature, day length (photoperiod), and elevated CO_2_. Our overall predictions of crop yield change were generally in agreement with other studies^9,17,19,20^.

### Carbon input trends

Crop yields are good indicators of total net primary production and are found to be proportional to the total C input to soils in the U.S. Corn Belt^21^ (i.e., crop yield to total biomass does not vary much geographically). Our simulations predicted the average input to be 3.7 Mg C ha^−1^ year^−1^ under the no climate change scenario for the 2041–2070 period (Supplementary Fig. 4). With climate change but no adaptation, all counties (an administrative subdivision of a state in the U.S.) had lower C input (GCMs ensemble mean) compared with the baseline. However, with adaptation, more than half of the counties (most counties in the northern part of the region) had higher C input than those of the baseline. Compared with the no adaptation scenario, all counties in the Corn Belt maintained higher C input with adaptation. The average C input across climate scenarios in the Corn Belt region was predicted to be 3.0 and 3.9 Mg C ha^−1^ (a change of − 19% and 5% from the baseline) with no adaptation and adaptation scenarios, respectively. The standard deviation of the adaptation scenarios across the three GCMs was 47% lower than without adaptation, suggesting a counteracting effect of adaptation to climate change.

### Decomposition factor trend

In contrast to C input, the decomposition factor, which reflected the relative change in decomposition rate due to temperature and moisture effect (ranges from 0 to 1, with higher rates associated with values closer to 1), increased in all counties regardless of the adaptation (Supplementary Fig. 4). Larger decomposition factors can be explained by an increase in soil temperature and wetter soil conditions associated with climate change projections. Wetter soil conditions were a result of a reduced transpiration rate under high levels of atmospheric CO_2_ and increased precipitation projected by the three GCMs. The average decomposition factor (across three GCMs) was predicted to be 0.41, 0.49, and 0.47 for baseline, without adaptation, and with adaptation scenarios. The standard deviations were 27% lower with adaptation for the decomposition factor across the three GCMs than without adaptation. If adaptation does not occur under the GCM scenarios, the growing period of the crops was reduced due to global warming and resulted in less total transpiration and wetter soil conditions, which increased decomposition. With adaptation, the longer-season maturity variety continued to grow over a longer period and consumed more water, reducing soil moisture to lower levels, thus lowering decomposition (average annual evapotranspiration was 2.0–5.1% higher in the adaptation scenario compared with no adaptation). The soil moisture differences among GCMs were lower with crop adaptation.

### Soil organic C trends

**Figure 2:**
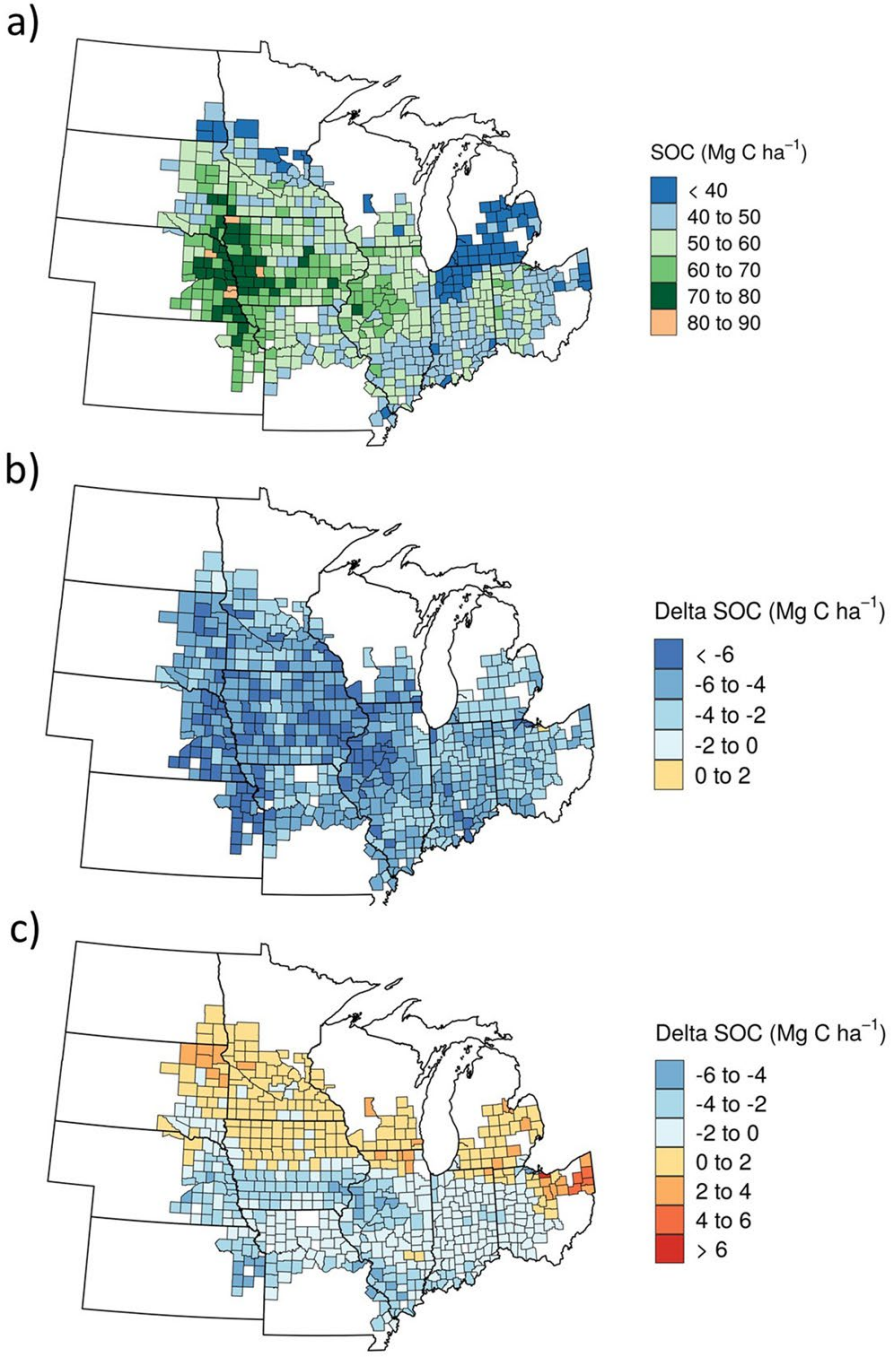
Predicted soil organic carbon (SOC) in the top soil (0–20 cm) averaged for the three GCMs in the U.S. Corn Belt, including (**a**) the baseline scenario and (**b**) the difference between the no adaptation scenario and baseline, and (**c**) the difference between the adaptation scenario and baseline. Maps were generated using the R “ggmap” package^39^ (Version 2.6.1; https://journal.r-project.org/archive/2013-1/kahle-wickham.pdf).

Without climate change (baseline scenario), the predicted sub-region SOC in the top soil ranged from less than 30 Mg C ha^−1^ to more than 80 Mg C ha^−1^(Fig. 2a). The highest levels of SOC were found in the western part of the region where soil clay content is high (Supplementary Fig. 1). The low levels of SOC found in Michigan and northern Indiana can be attributed to the soils with high sand content. In addition, tillage intensity varies among the counties^22^, contributing to differences in SOC levels among counties. With climate change, there were losses of SOC in almost all counties if there was no adaptation (Fig. 2b). This was the net result of decreased C inputs and increased decomposition rates (Supplementary Fig. 4). With adaptation, the northern part of the Corn Belt tended to gain SOC (Fig. 2c) due to increased C inputs (Supplementary Fig. 4), while the other areas tended to lose SOC. Compared with no adaptation, the adaptation scenario resulted in higher SOC stocks in all counties of the Corn Belt, which was also found in a modeling study of a site in Michigan^9^.

**Figure 3:**
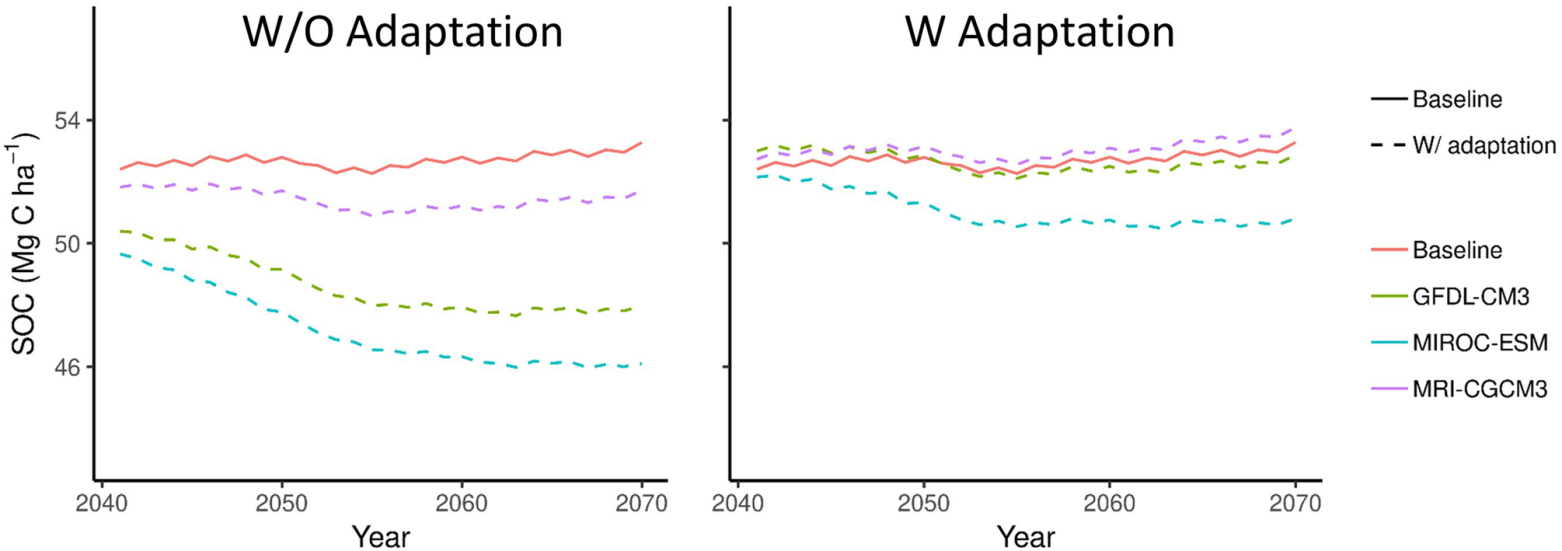
Area-weighted average soil organic carbon stocks for simulations with and without adaptation for corn/soybean rotations between 2041 and 2070. These projections are based on the same historical data and initial values for SOC pools.

Over the period from 2041 to 2070, the average SOC in the Corn Belt reached a new equilibrium state in the baseline scenario with no climate change (Fig. 3). Without adaptation in the climate change scenarios, SOC decreased over time. Rapid loss of C was predicted for the 2041–2050 period with the rate decreasing gradually over the 2051–2070 period. With adaptation, two GCMs (GFDL-CM3 and MRI-CGCM3) predicted similar changes over time as the baseline, while the SOC values for the other GCM (MIROC-ESM) were slightly lower than the baseline.

These results show that adaptation with the selection of longer-season varieties can lead to a similar SOC level as the baseline and GCM scenarios under a moderate climate change projection (RCP 4.5). Although the predicted climate was very different for the three GCMs, the SOC levels with adaptation were similar and not very different from the baseline with no climate change. In contrast, without adaptation, SOC storage levels were farther apart from each other and the baseline. This indicates a strong resistance to the effects of climate change in agricultural systems in the Corn Belt region if there is the selection for alternative varieties and planting dates that are better adapted to the changing climatic conditions.

We found the variation (CV 2.89%) of the predicted total SOC stock across the GCMs in the adaptation scenarios was much smaller than the variation (CV 5.88%) in the no adaptation scenarios. This corresponds to reduced variation in C input and decomposition factors. Because the increased decomposition rate was compensated by the higher overall C input, the SOC stock maintained similar levels as the baseline without climate change.

### Limitations

In this study, we did not evaluate the possibility of new technologies that could be developed in the future and influence production, decomposition and other variables influencing SOC levels, as these changes are difficult to predict. For example, new technologies associated with crop breeding and other genetic improvements, pest control, and other developments could increase C input and result in higher SOC stocks than our predictions. Management practices such as adding cover crops, which increases C input, may also further enhance SOC stocks^23^. In contrast, large areas of removal of corn stover for biofuel production (not considered in our simulation) could reduce the total SOC stock^24^. Although most SOC is concentrated in the surface soil, subsoil C has been found to respond to warming climates and affects SOC stocks^25^. Future research should also address subsoil C dynamics.

## Conclusions

The Corn Belt is the core area of U.S. crop production, and we found that management adaptation, which is often ignored in SOC studies, results in strong resistance to SOC losses that would otherwise occur with climate change. This resistance could enhance food security through its impact on soil health, fertility, and water holding capacity^26^. It also indicates a very low risk of losing SOC in this region, which will limit future carbon emissions to the atmosphere and further carbon debt from anthropogenic management of these lands^27^. However, there are counties in the southern part of the Corn Belt that are predicted to lose carbon even with adaptation. With future technology advancements and other management changes that are not considered in our study, it may be possible to reduce losses of SOC throughout the entire Corn Belt and potentially create a net carbon sink for atmospheric CO_2_ in the future^28^.

## Methods

The region of our simulation included 12 states in the U.S. Corn Belt (IA, IL, IN, KS, MI, MN, MO, ND, NE, OH, SD, and WI) (Supplementary Fig. 1).

### Climate change scenarios and climate data

Future weather data were generated using three General Circulation Models (GCMs) simulating the Representative Concentration Pathway (RCP) 4.5 greenhouse gas concentration trajectory^8^. Historical weather data used in the baseline scenario were derived from the 1980–2010 AgMERRA historical daily weather data^29^ (resampled at 32 km spatial resolution). GCM projections were downscaled to the AgMERRA spatial resolution and daily temporal resolution using the statistical method referred as “delta approach”^30^. The three GCMs used are MRI-CGM3^31^, MIROC-ESM^32^, and GFDL-CM3^33^. Supplementary Fig. 2 showed the characteristics of the three GCMs of the 2041–2070 period.

### DayCent model, data and model implementation

The biogeochemical DayCent model was used in our study. We simulated 7 scenarios of corn/soybean rotation for the period of 2011–2070, which were combinations of three climate change scenarios (three GCMs of RCP 4.5) and two adaptation scenarios. Additionally, we simulated a baseline with no climate change. In the adaptation scenario, crop planting dates and crop maturity groups for each location were changed every decade to optimize production given the climate change conditions from the GCMs. In the baseline scenario, we used historic weather data and a stable CO_2_ concentration of 389 ppm. In the adaptation scenario, CO_2_ concentration increased from 389 in 2010 to 524 by 2070^8^. The DayCent model was used to project SOC dynamics for 54,912 survey point locations that were in a corn/soybean rotation across the Corn Belt between 1990 and 2010. U.S. county boundaries were used to represent counties and all the data were summarized at the sub-region scale. Soil information was derived from the Soil Survey Geographic Database (SSURGO)^34^. More detailed information about the model and the regional simulation can be found in the Supplementary Materials.

Management data were derived from various sources or predicted. U.S. county-level tillage data (grouped into full, reduced, and no-tillage) were from the 2008 Crop Residue Management Survey conducted by the Conservation Technology Information Center (CTIC)^22^. Nitrogen fertilization rates were calculated using the predicted crop yield potential from DayCent for each scenario and recommended application rates to meet the targeted yields.

Planting dates for the baseline and no adaptation scenario were from the U.S. Department of Agriculture National Agricultural Statistics Service (NASS)^35^. To simulate the varieties of crops, we divided the crops into maturity groups (Supplemental Table 1). The spatial distributions of the maturity groups were derived from information provided by seed companies. The planting dates and maturity group distributions for each crop in the adaptation scenario were calculated based on a regression with temperature and estimated frost dates. Grain harvests were scheduled at the end of the growing season after maturity for corn and soybeans.

To establish a base SOC level (initial values for SOC pools) for each location, we conducted a model intialization^36^ using cropping histories from a variety of literature and historical databases developed for the US national greenhouse gas inventory^37^. In the pre-2011 historical simulation period, the radiation use efficiency (RUE) parameter was adjusted every 10 years from 1950 through 2010 to fit the simulated regional yield to the NASS reported historical yield trends for corn and soybean to represent crop breeding effects on production. Fertilizer rate was assumed to linearly increase from 1950 to 1980. Reduced till and no-till management was assumed to start in 1981 and only full tillage was used before 1981 in the historical simulations. The same initial values for SOC were used in each of the projections.

### Model parameterization and verification

The crop parameters used for each maturity group are shown in Supplementary Table S1. The parameters were either from published data or model calibration using the experimental sites (Supplementary Table 2). We evaluated the DayCent simulated crop yields with NASS reported data^13,38^. More information about model parameterization and verification can be found in the Supplementary Materials.

## Supplementary information

Supplementary Information.

## Data Availability

The datasets generated during and/or analyzed during the current study are available from the corresponding author on request. However, the NRI survey data and location information are not publicly accessible because the data contain confidential information as mandated by law, 7 USC 2276, and interpretive policy delineated in NRCS General Manual Title 290, Part 400.11, B(4).
