# Supplementary material for: Adaptation in U.S. Corn Belt increases resistance to soil carbon loss with climate change
Source: Sci Rep. 2020 Aug 14;10:13799. doi: 10.1038/s41598-020-70819-z (PMC7429838; doi:10.1038/s41598-020-70819-z)
Supplement: Supplementary file 1 — Supplementary Information. [file 41598_2020_70819_MOESM1_ESM.pdf]

Supplementary Information

Adaptation in U.S. Corn Belt Increases Resistance to Soil Carbon Loss with Climate Change

Yao Zhang, Ernie Marx, Stephen Williams, Ram Gurung, Stephen Ogle, Radley Horton, Daniel Bader, and Keith Paustian

Figures

a) NRI location count

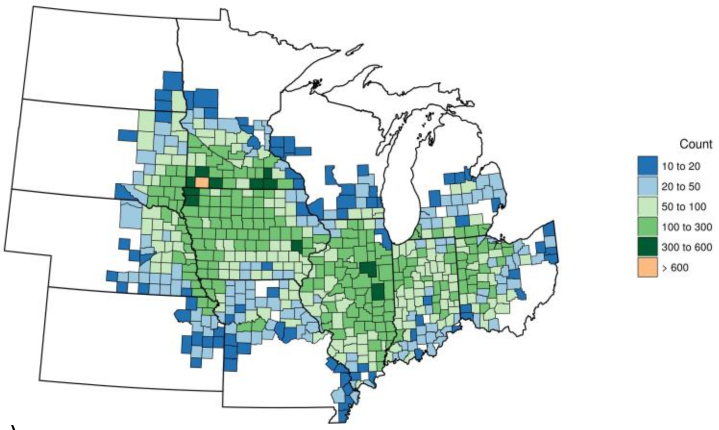

b) Clay Content

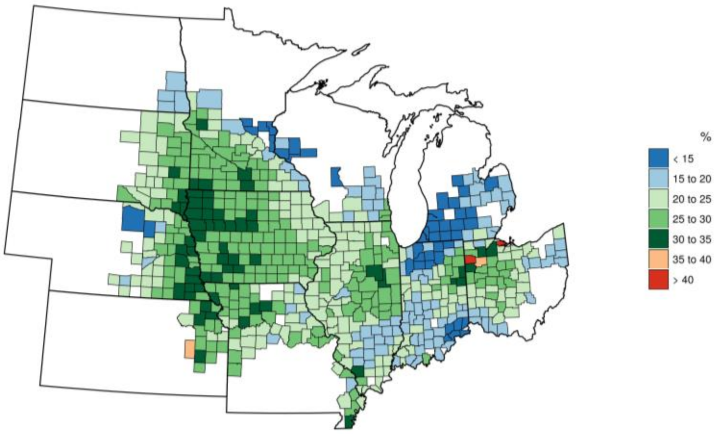

c) Sand Content

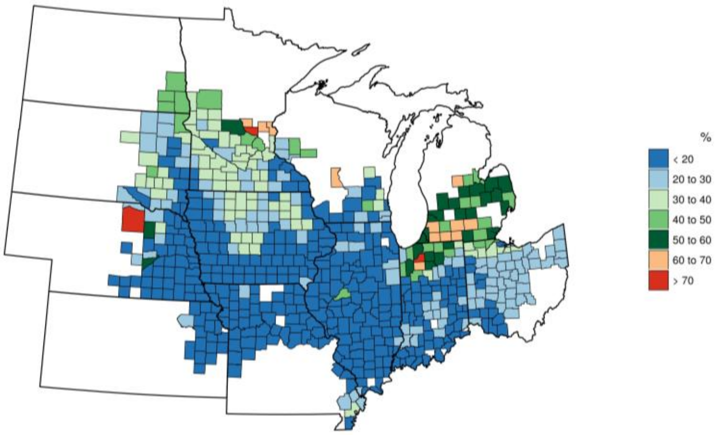

Figure S1. The maps of a) the number of NRI locations in corn/soybean rotation, b) the area-weighted average of clay content, and c) the area-weighted average of sand content in each sub-region. Maps were generated using the R "ggmap" package[1] (Version 2.6.1; http://journal.r-project.org/archive/2013-1/kahle-wickham.pdf).

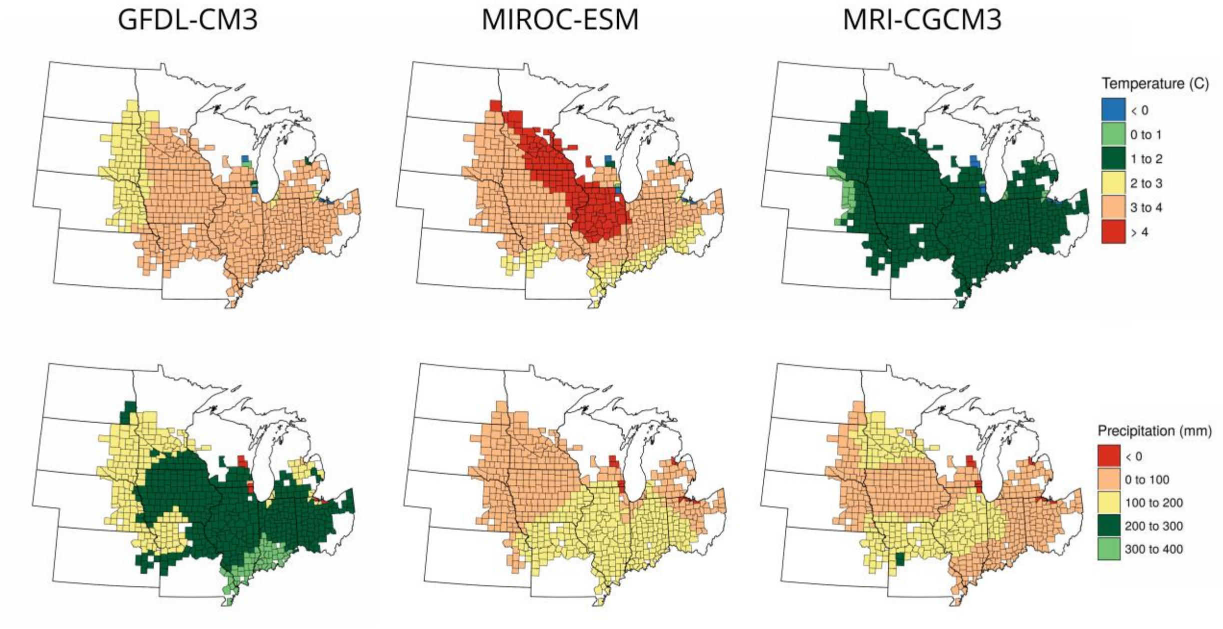

Figure S2. Changes in annual mean temperature and precipitation of the three GCMs (2041-2070 relative to 1981-2010). Maps were generated using the R "ggmap" package[1] (Version 2.6.1; http://journal.r-project.org/archive/2013-1/kahle-wickham.pdf).

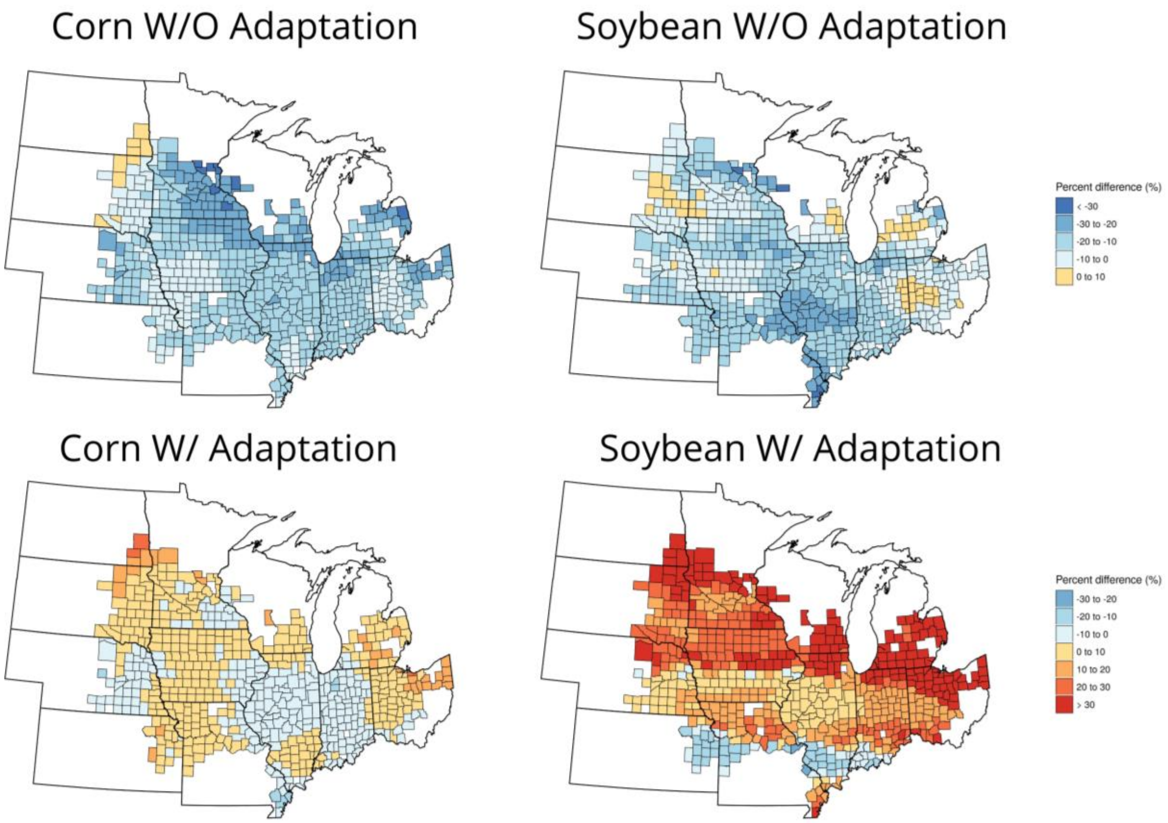

Figure S3. Crop grain yield of climate change scenarios (ensemble mean of three GCMs) compared with the baseline scenario (2041-2070). Maps were generated using the R "ggmap" package[1] (Version 2.6.1; http://journal.r-project.org/archive/2013-1/kahle-wickham.pdf).

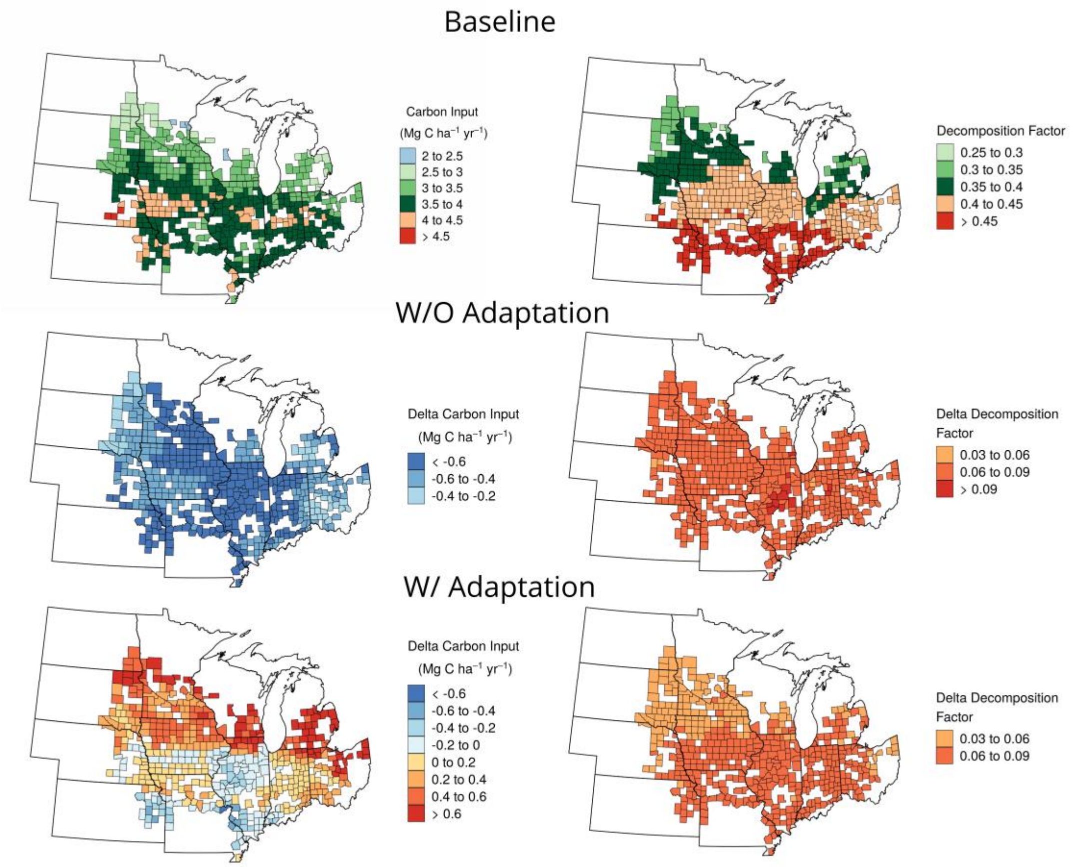

Figure S4. Predicted carbon input and decomposition factor (combined temperature and moisture effect) averaged for 3 GCMs for the baseline scenario (top row), the difference between no adaptation scenario and the baseline (second row), and the difference between the adaptation scenario and the baseline (third row). Maps were generated using the R "ggmap" package[1] (Version 2.6.1; http://journal.r-project.org/archive/2013-1/kahle-wickham.pdf).

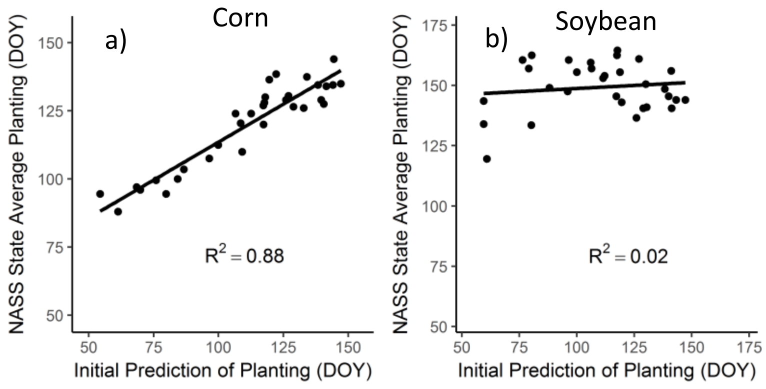

Figure S5. Correlation between the initial prediction of planting dates (DOY) using temperature and NASS reported average DOY for corn and soybean.

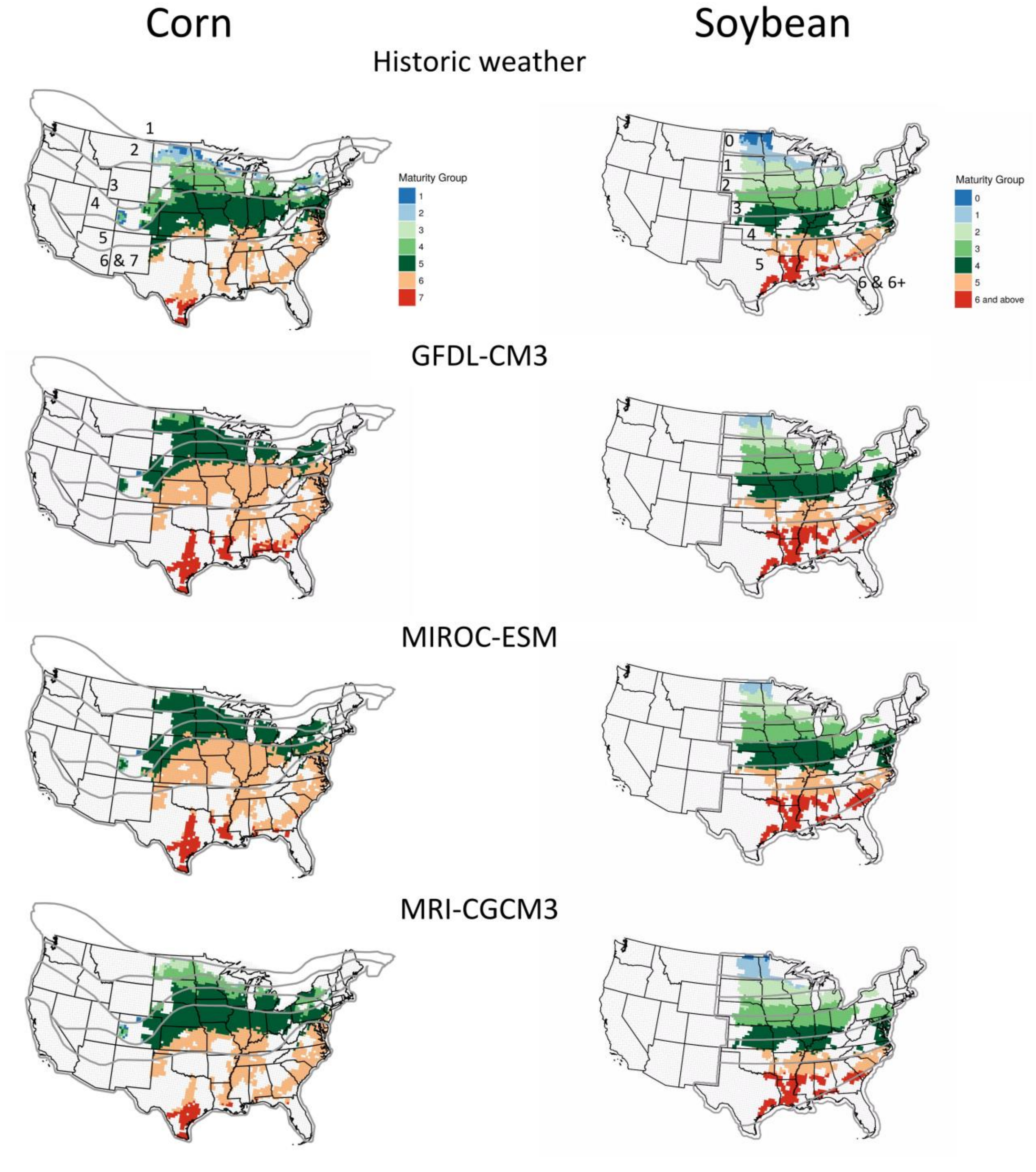

Figure S6. The predicted maturity groups for corn and soybean for the 2061-2070 decade. Gray boundaries represent crop maturity distributions reported by the seed companies and the notation numbers were the group numbers in Supplementary Table 1. Maps were generated using the R "ggmap" package[1] (Version 2.6.1; http://journal.r-project.org/archive/2013-1/kahle-wickham.pdf).

Table S1. Parameters used for each maturity group. The spatial distribution of maturity groups can be found in Figure S4. Growing degree days (GDD) and biological days (BD)[2] were used for corn and soybean, respectively.

	Corn							Soybean							
**Maturity group**	1	2	3	4	5	6	7	0	1	2	3	4	5	6	7
**Corn CRM/Soybean Maturity Group**	68-77	78-87	88-97	98-105	106-115	116-125	125+	00-0	I	II	III	IV	V	VI	VII
**GDD/BD to maturity**	1050	1150	1250	1350	1500	1600	1700	34	37	39	41	44	46	49	51
**GDD/BD to maximum GLAI**	600	600	700	700	800	850	900	18	18	19	20	20	21	22	22
**Critical photoperiod (hour)**	NA							14.1	13.8	13.6	13.4	13.1	12.8	12.8	12.3
**Photoperiod sensitivity**	NA							0.171	0.203	0.249	0.285	0.294	0.303	0.311	0.32
**Green leaf weight ratio at emergence**	0.90							0.95					0.85		
**Green leaf weight ratio at maximum GLAI**	0.25							0.35							
**Specific leaf area (m^2 g^{-1})**	0.02							0.025					0.03		
**Extinction coefficient**	0.55							0.50					0.45		
**Radiation use efficiency for total biomass production (g m^{-2} $langley^{-1}$ PAR)**	0.18							0.95							
**Initial biomass at emergence (g m^{-2})**	0.30							1.00					2.00		
**CO2 effect on plant production**	1.00							1.12							
**CO2 effect on transpiration**	0.82							0.58							

Table S2. Experimental sites used for calibration.

State	City	Latitude	Longitude	Records[a] of corn	Records of soybean	Data Source
CO	Fort Collins	40.7	-105.0	15	0	GRACEnet database[b]
IA	Ames	42.0	-93.8	4	0	GRACEnet database
KY	Bowling Green	36.9	-86.5	6	0	GRACEnet database
MN	Morris	45.7	-95.8	8	8	GRACEnet database
NE	Mead	41.2	-96.4	17	0	GRACEnet database
SD	Brookings	44.4	-96.8	5	5	GRACEnet database
AL	Shorter	32.4	-85.9	3	0	Causarano et al.[3]
IL	Bondville	40.0	-88.3	3	2	Zhang et al. [4]
NE	Mead	41.2	-96.5	26	10	Zhang et al. [4]
CO	Akron	40.2	-103.1	3	0	Zhang et al. [5]
CO	Fort Collins	40.7	-105.0	5	0	Zhang et al. [5]
CO	Greeley	40.5	-104.6	4	0	Zhang et al. [5]
MI	Hickory Corners	42.2	-85.24	8	9	Grandy et al. [6]
PA	Kutztown	40.6	-75.8	20	16	Hepperly et al. [7]
FL	Gainesville	29.6	-82.4	0	12	DSSAT database[c]
IW	Ames	42.0	-93.5	0	4	DSSAT database
MN	Morris	45.6	-95.7	0	1	DSSAT database
OH	Wooster	40.8	-81.9	0	2	DSSAT database
MO	Centralia	39.2	-92.2	0	3	Wang et al. [8]

a Records are year and treatment combinations.

b GRACEnet (Greenhouse gas Reduction through Agricultural Carbon Enhancement network) is a USDA-ARS research program[9].

c DSSAT database is within DSSAT crop model package [10]

Simulation Details

Regional Simulation Setup

The crop field locations were obtained from the National Resources Inventory (NRI) survey, which is a comprehensive long-term survey conducted by the U.S. Department of Agriculture's Natural Resources Conservation Service (NRCS) [11]. Management data such as crop rotation and irrigation are available in the survey. A total of 54,912 point locations were selected and corn/soybean rotation was modeled at each location. Locations were classified as predominantly corn-soybean if at least one-third of the years were cropped in corn and one third in soybeans over the recent history in the NRI survey.

In our simulation, we applied automatic irrigation in the DayCent simulations, if the fields were irrigated according to the survey. Tillage practices were modeled by grouping them into three categories: full tillage (FT; less than 15% residue remaining), reduced tillage (RT; 15-30% residue remaining), and no tillage (NT; above 30% residue remaining) using the data from the 2008 Crop Residue Management Survey conducted by the Conservation Technology Information Center (CTIC)[12].

Nitrogen fertilizer application rates were calculated in two steps. We first simulated the C dynamics using a routine that automatically adds adequate fertilizer to avoid nitrogen limitations. The fertilizer rates for the second simulation were calculated using the potential crop yield for each decade from the first simulation and recommended application rates from DuPont Pioneer to meet the targeted yield (1.2 lb/bu or 0.021 kg/kg for corn and 1.5 lb/bu or 0.025 kg/kg for yield above 60 bu or 1633 kg for soybean; https://www.pioneer.com/home/site/us/agronomy/library/nitrogen-fertilizer-for-soybean/).

Simulations were executed with customized scripts in R to automatically build DayCent input files, simulate the histories and projections, and analyze the outputs. Simulations were performed using a computer cluster with 440 processors. Simulation results were aggregated to the county level for analysis by weighting NRI points using the expansion factors from the NRI survey, which are based on the two-stage sampling design.

DayCent model

The DayCent model[13] is a widely used biogeochemical model for simulations of soil carbon, soil nitrogen, and crop growth[5,14,15]. The model was used to simulate corn and soybean yields for the contiguous U.S. and found to compare well with NASS reported yield values[16]. Two methods are available for modeling crop phenology. One is the growing degree day (GDD) method which is commonly used by seed companies for corn development (base temperature of 10 C˚ and ceiling temperature of 30 C˚). Another newly added method is the biological days (BD) method[2] which simulates the photoperiod effect on soybean phenology. The model simulates the elevated CO_2 effect on crop production, transpiration, root/shoot ratio, and carbon/nitrogen ratio[17-20].

Maturity groups, planting dates and maturity dates.

Our study region was the U.S. Corn Belt. We analyzed the current crop management for the entire U.S. in order to determine the adaptation of crops under climate change. To simulate the varieties, we

divided the crops into groups (Supplemental Table 1). The comparative relative maturity (CRM) is a common method of labeling the length of time to maturity for corn. Dupont Pioneer Seed Company grouped CRMs spatially (https://www.pioneer.com/home/site/us/agronomy/library/compare-maturity-corn-products/; accessed 05/06/2016). We adapted the map and associated the CRM with GDD[21]. For soybeans, we used the maturity group map from Monsanto (https://www.aganytime.com/asgrow/mgt/planting/Pages/Soybean-Maturity.aspx; accessed 05/06/2016); each maturity group was represented by the difference in their BD to maturity according to the sensitivity of the variety to temperature and photoperiod[2].

In the adaptation scenario, planting dates and maturity groups were set to change every decade in our simulation. We created a simple regression model to predict the planting dates by analyzing the relationship between air temperature and the NASS reported survey data for corn as corn planting dates are affected by temperature[22]. We first estimated the dates when temperature was not a restriction for planting based on the average weekly air temperature (threshold of 15.4 C) and the average last day of frost (daily minimum temperature below 0 C)[22] for all the major corn-growing counties in U.S. Note that counties south of the Corn Belt were also included in the development of regression as the climate in these areas are similar to the projected climate in the study region. These dates were compared to state average planting dates from NASS[23] to derive the regression equations (Supplementary Fig. 5a). A similar calculation was conducted for soybean planting dates. Even though the correlation for soybean was lower (Supplementary Fig. 5b), the RMSE of 10.2 days based on the regression model predictions demonstrated that the effect on grain yields were small for regional simulations. Soybean planting dates are not as strongly correlated with temperature as corn because soybean growth also depends on day length.

We also used different methods for corn and soybean to predict maturity groups. i.e., length of the growing season, for the climate change projections in the adaptation scenario. For corn, which is not affected by day length, we used the first day of frost in the fall/winter (decadal average) to approximate the maturity date. To avoid heat stress at critical development stages, farmers use hybrids that mature long before the first frost dates in regions that are south of the Corn Belt[24]. Therefore, we matched the maximum GDDs from planting to frost date to the reported GDDs across the US (Supplementary Tab. 1) and then applied these regression relationships for future predictions (Supplementary Fig. 6).

For soybeans, we developed a regression model to predict the physiological maturity (PM) dates. We used NASS reported planting dates and historical distribution of maturity group from data compiled by seed companies to calculate the PM dates for each weather grid cell (Supplementary Tab.1). These PM estimates were treated as observations and were compared to the predicted first day of frost in the fall/winter to develop the linear regression model for predicting PM dates (RMSE 9.3 days). For both the baseline and climate change scenarios, the BD values were calculated using the planting and PM dates. The suitable maturity group for a weather grid was determined for each decade by selecting the maturity group with BD (Supplementary Tab. 1) closest to the predicted BD in that weather grid (Supplementary Fig. 6).

Model Calibration and Verification

Details of model calibration and verification can be found in our earlier study[25]. Based on our previous sensitivity analysis[26], crop parameters were either derived from literature or calibrated using experimental sites located in different regions of the U.S. (Supplementary Tab. 2). Growing degree days for corn were calculated from a regression in a previous study[27]. Soybean photoperiod related parameters were taken directly from the work of Soltani and Sinclair[2]. The parameters associated with the effect of elevated CO_2 on production and water use were from our research[28]. The SOC decomposition parameters were from the U.S. greenhouse gas (GHG) inventory[29]. DayCent simulated corn and soybean yields in the contiguous U.S. with more accuracy than most other published studies using process-based models, with an overall R^2 of 0.54 and 0.54 for corn and soybean, respectively[25].

We did not assume any yield potential increase in the scenario simulations. Yields are likely to increase further due to technology development and other factors in the future[33]. However, it was beyond the scope of our study to predict the potential for future crop breeding and genetic improvements to increase the yield potentials for corn and soybeans. Our objective was to determine the role of crop adaptation alone on SOC dynamics with future climate change by parameterizing the model to use current crop varieties. Therefore, this analysis addresses the potential for adaptation through selection of varieties that currently exist in the United States. There may be even greater opportunity to enhance C input and increases in SOC in the future with crop breeding and other genetic improvementsin combination with evolving technologies and management strategies to maintain soil health and meet food security goals.

Supplementary References

1 Kahle, D. & Wickham, H. ggmap: Spatial Visualization with ggplot2. *R Journal* **5**, 144-161 (2013).

2 Soltani, A. & Sinclair, T. R. *Modeling Physiology of crop development, growth and yield*. (CABI International, 2012).

3 Causarano, H. J. *et al.* Simulating field-scale soil organic carbon dynamics using EPIC. *Soil Science Society of America Journal* **71**, 1174-1185, doi:10.2136/sssaj2006.0356 (2007).

4 Zhang, Y., Suyker, A. & Paustian, K. Improved crop canopy and water balance dynamics for agroecosystem modeling using DayCent. *Agronomy Journal* **110**, 511-524, doi:10.2134/agronj2017.06.0328 (2018).

5 Zhang, Y., Hansen, N., Trout, T., Nielson, D. & Paustian, K. Modeling Deficit Irrigation of Maize with the DayCent Model. *Agronomy Journal* **110**, 1754-1764, doi:10.2134/agronj2017.10.0585 (2018).

6 Grandy, A. S., Loecke, T. D., Parr, S. & Robertson, G. P. Long-term trends in nitrous oxide emissions, soil nitrogen, and crop yields of till and no-till cropping systems. *Journal of Environmental Quality* **35**, 1487-1495, doi:10.2134/jeq2005.0166 (2006).

7 Hepperly, P., Lotter, D., Ulsh, C. Z., Seidel, R. & Reider, C. Compost, Manure and Synthetic Fertilizer Influences Crop Yields, Soil Properties, Nitrate Leaching and Crop Nutrient Content. *Compost Science & Utilization* **17**, 117-126 (2009).

8 Wang, F., Fraisse, C. W., Kitchen, N. R. & Sudduth, K. A. Site-specific evaluation of the CROPGRO-soybean model on Missouri claypan soils. *Agricultural Systems* **76**, 985-1005, doi:10.1016/s0308-521x(02)00029-x (2003).

9 Del Grosso, S. J. *et al.* Introducing the GRACEnet/REAP Data Contribution, Discovery, and Retrieval System. *Journal of Environmental Quality* **42**, 1274-1280, doi:10.2134/jeq2013.03.0097 (2013).

10 Decision Support System for Agrotechnology Transfer Version 4.5. (University of Hawaii, Honolulu, HI., 2010).

11 USDA. Summary Report: 2007 National Resources Inventory. 123 (Natural Resources Conservation Service, Washington, DC, and Center for Survey Statistics and Methodology, Iowa State University, Ames, Iowa, 2009).

12 CTIC. Crop Residue Management Survey. (Conservation Technology Information Center, West Lafayette, Indiana, USA, 2008).

13 Parton, W. J., Hartman, M., Ojima, D. & Schimel, D. DAYCENT and its land surface submodel: description and testing. *Global and Planetary Change* **19**, 35-48, doi:10.1016/s0921-8181(98)00040-x (1998).

14 Robertson, A. D. *et al.* Climate Change Impacts on Yields and Soil Carbon in Row Crop Dryland Agriculture. *Journal of Environmental Quality* **47**, 684-694, doi:10.2134/jeq2017.08.0309 (2018).

15 Stehfest, E., Heistermann, M., Priess, J. A., Ojima, D. S. & Alcamo, J. Simulation of global crop production with the ecosystem model DayCent. *Ecological Modelling* **209**, 203-219, doi:10.1016/j.ecolmodel.2007.06.028 (2007).

16 Zhang, Y. *Simulating Canopy Dynamics, Productivity and Water Balance of Annual Crops from Field to Regional Scales . Ph.D. dissertation.* PhD thesis, Colorado State University, (2016).

17 Del Grosso, S. *et al.* (Natural Resource Ecology Laboratory, Colorado State University, Fort Collins, Colorado, 2015).

18 Metherell, A. K., Harding, L. A., Cole, C. V. & Parton, W. J. in *'Great Plains System Research Unit, Technical Report No. 4'.* (1993).

19 Luo, Y. Q. *et al.* Modeled interactive effects of precipitation, temperature, and CO2 on ecosystem carbon and water dynamics in different climatic zones. *Global Change Biology* **14**, 1986-1999, doi:10.1111/j.1365-2486.2008.01629.x (2008).
20 Lee, J., De Gryze, S. & Six, J. Effect of climate change on field crop production in California's Central Valley. *Climatic Change* **109**, 335-353, doi:10.1007/s10584-011-0305-4 (2011).
21 Kucharik, C. J. Evaluation of a Process-Based Agro-Ecosystem Model (Agro-IBIS) across the US Corn Belt: Simulations of the Interannual Variability in Maize Yield. *Earth Interactions* **7** (2003).
22 Sacks, W. J., Deryng, D., Foley, J. A. & Ramankutty, N. Crop planting dates: an analysis of global patterns. *Global Ecology and Biogeography* **19**, 607-620, doi:10.1111/j.1466-8238.2010.00551.x (2010).
23 NASS. Field Crops: Usual Planting and Harvesting Dates. (USDA, National Agricultural Statistics Service, 2010).
24 Kansas State University Agricultural Experiment Station and Cooperative Extension Service. Corn Production Handbook. (2007).
25 Zhang, Y. *et al.* DayCent Model Predictions of NPP and Grain Yields for Agricultural Lands in the Contiguous U.S. *Journal of Geophysical Research: Biogeosciences* **In press**.
26 Zhang, Y., Arabi, M. & Paustian, K. Analysis of parameter uncertainty in model simulations of irrigated and rainfed agroecosystems. *Environmental Modelling and Software* (Under review).
27 Kucharik, C. J. Evaluation of a Process-Based Agro-Ecosystem Model (Agro-IBIS) across the US Corn Belt: Simulations of the Interannual Variability in Maize Yield. *Earth Interactions* **7**, 1-33 (2003).
28 Kent, J. & Paustian, K. in *AGU Fall Meeting Abstracts.*
29 US EPA. Inventory of U.S. Greenhouse Gas Emissions and Sinks: 1990-2014. (US Environmental Protection Agency (EPA), Office of Atmospheric Programs, Washington, DC., 2015).
30 NASS. USDA–National Agricultural Statistics Service (NASS) Quick stats. Available at http://quickstats.nass.usda.gov/. (2017).
31 Ruane, A. C., Goldberg, R. & Chryssanthacopoulos, J. Climate forcing datasets for agricultural modeling: Merged products for gap-filling and historical climate series estimation. *Agricultural and Forest Meteorology* **200**, 233-248, doi:10.1016/j.agrformet.2014.09.016 (2015).
32 Zhang, Y. & Paustian, K. Sensitivity of predicted agro-ecosystem variables to errors in weather input data. *Transactions of the ASABE*, In press (2019).
33 Petersen, L. K. Impact of Climate Change on Twenty-First Century Crop Yields in the U.S. *Climate* **7**, doi:10.3390/cli7030040 (2019).
